# Infectious Complications during Tandem High-Dose Chemotherapy and Autologous Stem Cell Transplantation for Children with High-Risk or Recurrent Solid Tumors

**DOI:** 10.1371/journal.pone.0162178

**Published:** 2016-09-14

**Authors:** Young Bae Choi, Eun Sang Yi, Ji-Man Kang, Ji Won Lee, Keon Hee Yoo, Yae-Jean Kim, Ki Woong Sung, Hong Hoe Koo

**Affiliations:** 1 Department of Pediatrics, Chung-Ang University Hospital, Seoul, Korea; 2 Department of Pediatrics and Samsung Medical Center, Sungkyunkwan University School of Medicine, Seoul, Korea; Chang Gung University, TAIWAN

## Abstract

We retrospectively analyzed infectious complications during tandem high-dose chemotherapy and autologous stem cell transplantation (HDCT/auto-SCT) in children and adolescents with high-risk or recurrent solid tumors. A total of 324 patients underwent their first HDCT/auto-SCT between October 2004 and September 2014, and 283 of them proceeded to their second HDCT/auto-SCT (a total of 607 HDCT/auto-SCTs). During the early transplant period of 607 HDCT/auto-SCTs (from the beginning of HDCT to day 30 post-transplant), bacteremia, urinary tract infection (UTI), respiratory virus infection, and varicella zoster virus (VZV) reactivation occurred in 7.1%, 2.3%, 13.0%, and 2.5% of HDCT/auto-SCTs, respectively. The early transplant period of the second HDCT/auto-SCT had infectious complications similar to the first HDCT/auto-SCT. During the late transplant period of HDCT/auto-SCT (from day 31 to 1 year post-transplant), bacteremia, UTI, and VZV reactivation occurred in 7.5%, 2.5%, and 3.9% of patients, respectively. Most infectious complications in the late transplant period occurred during the first 6 months post-transplant. There were no invasive fungal infections during the study period. Six patients died from infectious complications (4 from bacterial sepsis and 2 from respiratory virus infection). Our study suggests that infectious complications are similar following second and first HDCT/auto-SCT in children.

## Introduction

High-dose chemotherapy and autologous stem cell transplantation (HDCT/auto-SCT) has improved the outcomes of patients with high-risk solid tumors [1–3]. Infectious complications during HDCT/auto-SCT are a major cause associated with treatment-related morbidity and mortality [4,5]. Despite advances in infection prophylaxis and treatment during HDCT/auto-SCT [6–9], many clinical problems including the emergence of multi-drug-resistant (MDR) bacteria [10], the risk of *Clostridium difficile* infection [11], and the high incidence of bloodstream infections [12,13] still require further research.

Recently, an increasing number of studies have used tandem HDCT/auto-SCT [14–16], and their findings suggest that further dose intensification might improve the outcomes of patients with high-risk or recurrent tumors. However, the number of CD34^+^ cells for each session of tandem HDCT/auto-SCT could be lower than in single HDCT/auto-SCT because about half of the collected stem cells are infused during each HDCT/auto-SCT session. In addition, the second HDCT/auto-SCT could require a longer time for hematologic recovery than the first HDCT/auto-SCT, even when similar numbers of CD34^+^ cells are infused [17]. These findings suggest that infectious complications might be more frequent or severe in the second HDCT/auto-SCT than in the first, but studies addressing infectious complications during tandem HDCT/auto-SCT have been limited. Accordingly, we investigated infectious complications during tandem HDCT/auto-SCT.

## Materials and Methods

### Ethics Statement

This study has been reviewed and approved by the Institutional Review Board of Samsung Medical Center and informed consent was waived by the board. Patient records/information were anonymized and de-identified prior to analysis.

### Patients

We retrospectively reviewed the medical records of children and adolescents with high-risk or recurrent solid tumors who underwent their first HDCT/auto-SCT between October 2004 and September 2014 at Samsung Medical Center. A period from October 2004 to December 2009 and a period from January 2010 to September 2014 were defined as early and late study period, respectively. A period from the beginning of HDCT to day 30 post-transplant and a period from day 31 to 1 year post-transplant or the day of treatment-related mortality, tumor relapse/progression, or death, whichever occurred first were defined as early and late transplant period, respectively.

### Definitions

Fever was defined by a single axillary temperature ≥38°C. Bacteremia was defined as isolation of a bacterial pathogen from at least one set of blood cultures (one aerobic and one anaerobic) drawn from a patient. For coagulase-negative staphylococci and corynebacteria, isolation from at least two sets of blood cultures with the same antibiogram was required. Septic shock was defined as bacteremia with hypotension or the use of inotropic agents. Urinary tract infection (UTI) was defined by urinary symptoms and/or a fever and a hospital record of a positive urine culture. A positive urine culture was defined as the growth of ≥10^5^ colony-forming unit/mL of a single organism in midstream or bag urine specimens. *Clostridium difficile* infection (CDI) required the presence of diarrhea (defined as ≥3 unformed stools in 24 hours) or radiographic evidence of ileus or toxic megacolon and a positive stool test result for toxigenic *Clostridium difficile* or its toxins, or colonoscopic or histopathologic findings demonstrating pseudomembranous colitis [18]. Any Gram-negative isolate that exhibited resistance to at least two antibiotics used in empirical therapy (third and fourth-generation cephalosporins, carbapenems or piperacillin-tazobactam) was defined as MDR. From November 2008, patients with fever, respiratory symptoms or chest radiologic abnormalities were tested by the Seeplex multiplex polymerase chain reaction assay (Seegene Inc., Seoul, Korea) for 6 respiratory viruses (RVs): rhinovirus (RhV), respiratory syncytial virus (RSV), coronavirus (CoV), parainfluenza virus (PIV), adenovirus (AdV), and influenza virus (IV). RVs were categorized into 2 groups according to the symptomatology and clinical outcome. The low-risk RV group included RhV and CoV, and the high-risk RV group included RSV, PIV, AdV, and IV. Cytomegalovirus (CMV) disease was diagnosed according to the published recommendations [19]. Invasive fungal infection (IFI) was defined with the criteria set by the European Organization for Research and Treatment of Cancer/Invasive Fungal Infections Cooperative Group [20].

### Use of Antibacterial and Antifungal Agents

Cefepime was used as the first-line antibacterial agent, and teicoplanin and amikacin were added as second-line agents if fever persisted for three days on cefepime, or if a fever recurred despite more than three days of cefepime treatment. The antibiotics were changed to teicoplanin and imipenem (≥12 years of age) or meropenem (<12 years of age) as third-line agents for patients who had persistent neutropenic fever for an additional three days or for recurrent fever after treatment with second-line antibiotics for more than three days. From June 2011 onwards, we changed the antibiotic strategy; cefepime was the first-line agent, teicoplanin and cefepime were used for second-line therapy, and teicoplanin and meropenem were used as third-line agents. From October 2004 to April 2008, antifungal agents were used prophylactically or empirically and no antifungal agent was used in some patients. However, all patients received antifungal prophylaxis (fluconazole, itraconazole, or micafungin) after May 2008. All antibiotics including antifungal agents were discontinued after three consecutive days of no significant fever (<37.5°C), no evidence of documented or clinically suspected infection, and an absolute neutrophil count exceeding 0.5 × 10^9^/L, with exception of prophylactic trimethoprim-sulfamethoxazole for *Pneumocystis jiroveci*. Prophylactic acyclovir (from day ‒1 to the day of engraftment) was administered to prevent VZV reactivation between October 2004 and June 2005, but has not been used since July 2005 to minimize drug toxicity during HDCT.

### Statistical Analysis

To analyze the association between the incidence of infectious complications and potential clinical or laboratory risk factors, the Chi-square test or the Fisher’s exact test were used for binary variables, and the Mann-Whitney U test or Kruskal-Wallis test were used for continuous variables. Multivariable analysis was performed using linear regression analysis and binary logistic regression tests to examine the factors associated with fever duration and bacteremia. All values were 2-sided and statistical significance was accepted at the level of p < 0.05.

## Results

### Patient Characteristics

A total of 324 patients (196 males and 128 females) underwent their first HDCT/auto-SCT, and 283 of them proceeded to their second HDCT/auto-SCT (a total of 607 HDCT/auto-SCTs); however, the remaining 41 patients could not proceed to the second HDCT/auto-SCT due to tumor progression (n = 14), off-treatment (n = 11), treatment-related mortality (n = 10), follow-up loss (n = 3), subsequent allogeneic transplantation (n = 2), or parental refusal (n = 1). Transplantation characteristics are presented in Table 1. The most common diagnosis was neuroblastoma, followed by brain tumors. The most common tandem HDCT regimens were CEC (carboplatin + etoposide + cyclophosphamide) followed by MIBG-TM (^131^I-metaiodobenzylguanidine treatment + thiotepa + melphalan) for neuroblastoma, and CTE (carboplatin + thiotepa + etoposide) followed by the CyM (cyclophosphamide + melphalan) for brain tumors.

**Table 1:** Transplantation characteristics.

Characteristics	n = 607
**Median age at transplantation in months (range)**	58 (7–329)
**Diagnosis**	
**Neuroblastoma**	265 (43.6%)
**Brain tumors**	241 (39.7%)
**Bone and soft tissue sarcomas**	43 (7.1%)
**Retinoblastoma**	34 (5.6%)
**Wilms tumor**	18 (3.0%)
**Germ cell tumors**	6 (1.0%)
**HDCT regimens**	
**First HDCT (n = 324)**	
**CTE**	162 (50.0%)
**CEC**	149 (46.0%)
**Others**	13 (4.0%)
**Second HDCT (n = 283)**	
**CyM**	142 (50.2%)
**TM ± TBI (or** ^**131**^**I-MIBG)**	129 (45.6%)
**Others**	12 (4.2%)
**TBI and/or thiotepa-containing regimens**	344 (56.7%)

HDCT, indicates high-dose chemotherapy; CTE, carboplatin + thiotepa + etoposide; CEC, carboplatin + etoposide + cyclophosphamide; CyM, cyclophosphamide + melphalan; TM, thiotepa + melphalan; TBI, total body irradiation; ^131^I-MIBG, ^131^I-metaiodobenzylguanidine.

### Fever Duration during the Early Transplant Period

Fever occurred during the early transplant period in 508 (83.7%) of 607 HDCT/auto-SCTs for a median duration of 4 days (range 0‒27). In the multivariable analysis, inclusion of total body irradiation (TBI) and/or thiotepa in the HDCT regimen, which frequently causes severe mucositis, was associated with longer duration of fever. Otherwise, there was no difference in fever duration with respect to various clinical factors (Table 2). Interestingly, CD34^+^ cell number did not affect the fever duration, despite the difference in the time for neutrophil recovery according to the CD34^+^ cell number.

**Table 2:** Univariate and multivariate analysis for factors affecting fever duration during the early transplant period of tandem HDCT/auto-SCT.

Potential risk factors	Univariate analysis			Multivariate anaylsis		
	t-score	95% CI	p-value	t-score	95% CI	p-value
**Second HDCT/auto-SCT (compared to the first)**	–1.84	-1.20~0.04	0.067	–1.79	-1.17~0.05	0.073
**Inclusion of TBI and/or thiotepa in HDCT regimen**	3.96	0.62~1.85	<0.001	4.00	0.63~1.86	<0.001
**Infused CD34**^**+**^ **cells <2 × 10**^**6**^**/kg**	1.15	-0.31~1.18	0.249	1.07	-0.33~1.14	0.283
**Age at transplantation >100 months**	-1.09	-1.3~0.30	0.278	-1.11	-1.03~0.29	0.269

HDCT/auto-SCT, high-dose chemotherapy and autologous transplantation; CI, confidence interval; TBI, total body irradiation.

### Bacterial Infection during the Early Transplant Period

A total of 68 (11.2%) episodes of bacterial infections (35 in the first HDCT/auto-SCT and 33 in the second HDCT/auto-SCT) were documented during the early transplant period of 607 HDCT/auto-SCTs. These infections included 43 (7.1%) episodes of bacteremia, 14 (2.3%) episodes of UTI, and 11 (1.8%) episodes of CDI. Of 43 episodes with bacteremia, 33 were caused by Gram-negative organisms and 13 by Gram-positive organisms. A total of 10 septic shock episodes occurred, which were caused by 9 Gram-negative bacteremias and 1 Gram-positive bacteremia. Three patients died from bacterial sepsis during the early transplant period of HDCT/auto-SCT. All UTIs were caused by Gram-negative bacteria. Table 3 shows multivariate analysis for risk factors of bacteremia in the early transplant period of HDCT/auto-SCT. While inclusion of TBI and/or thiotepa in the HDCT regimen and lower number of CD34^+^ cells (<2 ± 10^6^/kg) were associated with more frequent Gram-positive bacteremia, older age (>100 months of age) was associated with more frequent Gram-negative bacteremia.

**Table 3:** Multivariate analysis for risk factors of bacteremia during the early transplant period of tandem HDCT/auto-SCT.

Potential risk factors	Gram-positive bacteremia			Gram-negative bacteremia		
	OR	95% CI	p-value	OR	95% CI	p-value
**Second HDCT/auto-SCT (compared to the first)**	0.72	0.21~2.49	0.604	0.67	0.32~1.40	0.286
**Inclusion of TBI and/or thiotepa in HDCT regimen**	6.28	1.34~29.50	0.020	0.60	0.29~1.26	0.178
**Infused CD34**^**+**^ **cells <2 × 10**^**6**^**/kg**	3.35	1.02~10.98	0.046	1.40	0.63~3.12	0.416
**Age at transplantation >100 months**	0.93	0.26~3.29	0.911	2.56	1.25~5.24	0.010

OR, odds ratio; CI, confidence interval; HDCT/auto-SCT, high-dose chemotherapy and autologous transplantation; TBI, total body irradiation.

### Viral Infection during the Early Transplant Period

**Fig 1:**
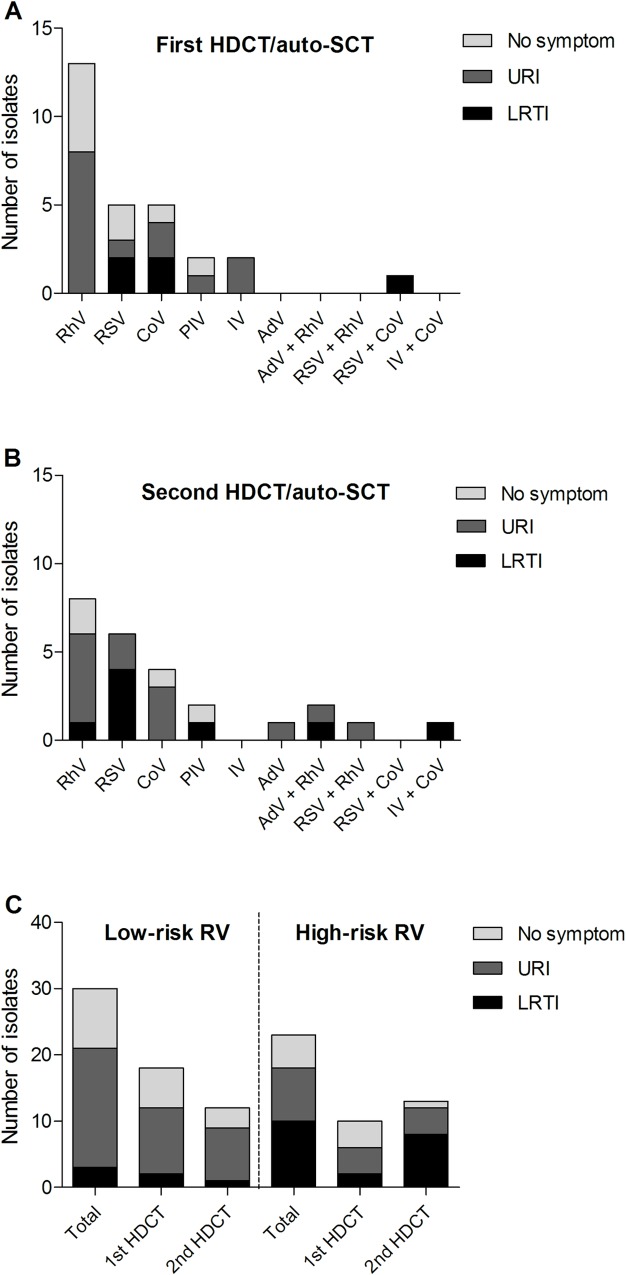
Respiratory virus infections during the early transplant period of tandem HDCT/auto-SCT. Number of isolates and respiratory symptoms for each respiratory virus during the first (A) and second (B) HDCT/auto-SCT. (C) The high-risk RV group had a higher incidence of LRTI than the low-risk RV group (43.5% versus 10.0%, p = 0.005). HDCT/auto-SCT, high-dose chemotherapy and autologous stem cell transplantation; URI, upper respiratory infection; LRTI, lower tract respiratory infection; RhV, rhinovirus; RSV, respiratory syncytial virus; CoV, coronavirus; PIV, parainfluenza virus; IV, influenza virus; AdV, adenovirus; RV, respiratory virus.

A total of 53 (13.0%) RV infection episodes were documented during the early transplant period of 409 HDCT/auto-SCTs from November 2008 to September 2014. There were 21 episodes caused by RhV, 11 by RSV, 9 by CoV, 4 by PIV, 2 by IV, 1 by AdV, and 5 by co-infections (AdV/RhV in 2, RSV/RhV in 1, RSV/CoV in 1, and IV/CoV in 1). Of 53 RV infections, upper respiratory infection (URI) developed in 26 (49.1%), lower respiratory tract infection (LRTI) in 13 (24.5%), and no respiratory symptoms developed in 14 (26.4%) cases. Detected RVs and clinical characteristics during tandem HDCT/auto-SCT are summarized in Fig 1A and 1B. The incidence of LRTI was higher in the high-risk RV group than in the low-risk RV group (43.5% versus 10.0%, p = 0.005, Fig 1C). Two patients died from RSV and AdV pneumonia. VZV reactivation occurred in 15 (2.5%) HDCT/auto-SCTs, which included 11 localized zoster, 3 disseminated zoster without visceral involvement, and 1 varicella. CMV disease developed in 2 (0.3%) patients, which included retinitis and enterocolitis.

From May 2010, all patients were screened for the presence of RV infection before HDCT/auto-SCTs (n = 293) and only RV-negative cases proceeded to the HDCT/auto-SCT. However, 14 RV-positive patients without respiratory symptoms proceeded to the HDCT/auto-SCT, because RV did not disappear during the waiting period. Eight (57.1%) of them experienced clinical respiratory disease (LRTI in 2 and URI in 6) during HDCT/auto-SCT without mortality. For the remaining 279 patients who were RV-negative prior to HDCT/auto-SCT, RV infection developed in 26 (9.3%) patients and 19 (6.8%) of them experienced clinical respiratory disease (LRTI in 5 and URI in 14).

### Invasive Fungal Infection during the Early Transplant Period

An antifungal agent was administered prophylactically or empirically in 512 (84.3%) and 49 (8.1%) HDCT/auto-SCTs, respectively. There were no cases of proven or probable IFI during the early transplant period of HDCT/auto-SCT. Fever duration was not different among patients receiving different antifungal treatment.

### Difference in Infectious Complications between the First and Second HDCT/Auto-SCT

There were no differences in transplant characteristics between the first and second HDCT/auto-SCT except higher use of TBI-containing regimens and delayed platelet recovery in the second HDCT/auto-SCT (Table 4). The incidence of fever and the proportion of patients using second-line antibiotics were higher in the first HDCT/auto-SCT than in the second HDCT. Fever duration was also longer in the first HDCT with borderline significance. Otherwise, there was no significant difference in infection parameters between the first and second HDCT/auto-SCT.

**Table 4:** Differences in infectious complications during the early transplant period of tandem HDCT/Auto-SCT.

Parameters	First HDCT/auto-SCT(n = 324)	Second HDCT/auto-SCT(n = 283)	p-value
**HDCT regimens**			
**TBI-containing regimen**	3 (0.9%)	49 (17.3%)	<0.001
**Thiotepa-containing regimens**	163 (50.3%)	129 (47.6%)	0.245
**Infused CD34**^**+**^ **cells (× 10**^**6**^**/kg)**	4.12 (0.29–220.20)	4.34 (0.29–157.60)	0.804
**Hematologic recovery**			
**Time (days) to reach an ANC 0.5 × 10**^**9**^**/L**^a^	10 (7–35)	10 (7–127)	0.537
**Time (days) to reach a PLT count 20 × 10**^**9**^**/L**^b^	21 (7–252)	26 (0–1,318)	<0.001
**≥ 1 episode of fever (≥ 38.0**^**°**^**C)**	289 (89.2%)	216 (76.3%)	<0.001
**Fever (≥ 38.0**^**°**^**C) duration (days)**	5 (0–23)	4 (0–27)	0.068
**Application antibiotics**			
**First-line antibiotics**	323 (99.7%)	278 (98.2%)	0.102
**Second-line antibiotics**	279 (86.1%)	223 (78.8%)	0.017
**Third-line antibiotics**	176 (54.3%)	144 (50.9%)	0.397
**Duration of antibacterial agents (days)**	14 (4–39)	13 (0–35)	0.140
**Bacteremia**	24 (7.4%)	19 (6.7%)	0.740
**Gram-positive bacteremia**	7 (2.2%)	6 (2.1%)	0.973
**Gram-negative bacteremia**	20 (6.2%)	13 (4.6%)	0.392
**Proven or probable invasive fungal infection**	0	0	1.000
**Urinary tract infection**	7 (2.2%)	7 (2.5%)	0.798
**Respiratory virus infection**^c^	28/213 (13.1)	25/196 (12.8%)	0.907
***Clostridium difficile* infection**	4 (1.2%)	7 (2.5%)	0.254
**CMV disease**	1 (0.3%)	1 (0.4%)	0.924
**Deaths associated with infectious complications**	3 (0.9%)	2 (0.7%)	1.000

HDCT/auto-SCT, high-dose chemotherapy and autologous stem cell transplantation; ANC, absolute neutrophil count; PLT, platelet; CMV, cytomegalovirus.

^a^ The first day that ANC exceeded 0.5 × 10^9^/L for 3 consecutive days.

^b^ The first day that PLT count exceeded 20 × 10^9^/L without transfusion for 7 days.

^c^ Results in 409 HDCT/auto-SCT from November 2008 to September 2014.

### Infectious Complications during the Late Transplant Period

A total of 281 of 283 patients who remained event free for more than 30 days after the second HDCT/auto-SCT were evaluated for infectious complications during the late transplant period of HDCT/auto-SCT. A total of 38 patients (13.5%) experienced 42 episodes of infection other than RV infection during the late transplant period. These infections included 21 (7.5%) episodes of bacteremia, 7 (2.5%) episodes of UTI, 2 (0.7%) episodes of CDI, 11 (3.9%) episodes of VZV infection (10 localized zoster and 1 varicella), and 1 (0.4%) episode of CMV disease. There were no cases of IFI and *Pneumocystis jiroveci* pneumonia. Most infectious complications during the late transplant period occurred during the first 6 months after HDCT/auto-SCT and were comprised primarily of bacteremia (14/21, 66.7%), VZV reactivation (8/11, 72.7%), and UTI (5/7, 71.4%). Of 21 episodes of bacteremia, 17 were caused by Gram-positive organisms and 4 by Gram-negative organisms. Eighteen of 20 patients who developed 21 episodes of bacteremia had indwelling central catheters at the time of infection. One patient who had suffered severe hepatic veno-occlusive disease and had been dependent on a mechanical ventilator at the time of infection died from bacterial sepsis during the late transplant period.

### Emergence of MDR Bacteria

A total of 114 (48 Gram-positive and 66 Gram-negative) bacteria were isolated during both the early and late study period of HDCT/auto-SCT (Table 5). Of 66 Gram-negative bacteria, 13 (19.7%) were MDR bacteria and the proportion of MDR bacteria changed from 13.3% during the early study period (2004–2009) to 25.0% during the late study period (2010–2014). The most common MDR Gram-negative bacteria was extended-spectrum-beta-lactamase (ESBL)-producing *Escherichia coli*, and its incidence changed from 2.2% during the early study period to 7.4% during the late study period. All 4 deaths from bacterial sepsis (2 in the early and 2 in the late study period) were caused by MDR bacteria: *Stenotrophomonas maltophilia* (n = 1), carbapenem-resistant *Acinetobacter baumannii* (n = 2), and ESBL-producing *Escherichia coli* (n = 1).

**Table 5:** Etiology of bacterial infection.

Pathogen category	Whole study period 2004–2014 (n = 114)	Early study period 2004–2009 (n = 46)	Late study period 2010–2014 (n = 68)
**Gram-positive bacteria**	48 (42.1%)	16 (34.8%)	32 (47.1%)
***Staphylococcus aureus***			
**Methicillin-resistant**	5 (4.4%)	3 (6.5%)	2 (2.9%)
**Methicillin-susceptible**	3 (2.6%)	0	3 (4.4%)
***Bacillus cereus* group**	8 (7.0%)	3 (6.5%)	5 (7.4%)
***Streptococcus* species**	4 (3.5%)	1 (2.2%)	3 (4.4%)
***Enterococcus* species**	4 (3.5%)	2 (4.3%)	2 (2.9%)
***Clostridium difficile***	13 (11.4%)	3 (6.5%)	10 (14.7%)
**Others**	11 (9.6%)	4 (8.7%)	7 (10.3%)
**Gram-negative bacteria**	66 (57.9%)	30 (65.2%)	36 (52.9%)
***Escherichia coli***			
**ESBL-positive**^**a**^	6 (5.3%)	1 (2.2)	5 (7.4%)
**ESBL-negative**	16 (14.0%)	4 (8.7)	12 (17.6%)
***Klebsiella pneumonia***			
**ESBL-positive**^**a**^	3 (2.6%)	0	3 (4.4%)
**ESBL-negative**	14 (12.3%)	8 (17.4%)	6 (8.8%)
***Pseudomonas aeruginosa***	5 (4.4%)	4 (8.7%)	1 (1.5%)
***Acinetobacter baumannii***			
**MDR**^**a**^	2 (1.8%)	1 (2.2%)	1 (1.5%)
**Non-MDR**	1 (0.9%)	0	1 (1.5%)
***Sphingomonas paucimobilis***^**a**^	1 (0.9%)	1 (2.2%)	0
***Stenotrophomonas maltophilia***^**a**^	1 (0.9%)	1 (2.2%)	0
***Enterobacter* species**	1 (0.9%)	1 (2.2%)	0
**Others**	16 (14.0%)	9 (19.6%)	7 (10.3%)

ESBL, extended-spectrum beta-lactamase; MDR, multi-drug-resistant.

^a^ Multi-drug resistant Gram-negative bacilli.

## Discussion

Studies addressing the infectious complications associated with tandem HDCT/auto-SCT for children and adolescents are limited. This study evaluated infectious complications in 324 patients with high-risk solid tumors, 283 of whom underwent tandem HDCT/auto-SCT, over a 10-year period in a single institue. To the best of our knowledge, this is the largest cohort of children and adolescents who underwent tandem HDCT/auto-SCT. In general, the frequency and severity of infectious complications in our cohort were similar to those in a cohort of 320 children and adolescents at the St. Jude Children’s Research Hospital (SJCRH), most of whom underwent single HDCT/auto-SCT [21]. We initially hypothesized infectious complications might be more frequent or severe in the second HDCT/auto-SCT than in the first HDCT/auto-SCT. In contrast, we found that episodes of fever were more frequent and the proportion of cases using second-line antibiotics was higher in the first HDCT/auto-SCT than in the second. Otherwise, there was no difference in infection parameters between the first and second HDCT/auto-SCT. In the multivariate analysis for risk factors of infectious complications during tandem HDCT/auto-SCT, inclusion of TBI and/or thiotepa which frequently causes severe mucositis, was a significant high-risk factor; however, the second HDCT/auto-SCT (compared to the first HDCT/auto-SCT) was not a significant factor. Collectively, these findings suggest that the second HDCT/auto-SCT does not increase the severity of infectious complications compared to the first, and that the severity of infectious complications during tandem HDCT/auto-SCT might be associated with HDCT regimens rather than the number of HDCTs.

In our study, the overall incidence of bacteremia during the early transplant period after HDCT/auto-SCT was 7.1%, which was lower than results from adult studies [13,22] and similar to the 7% incidence of overall bacteremia seen 0–30 days post-transplant in a SJCRH cohort [21]. This incidence of bacteremia might be acceptable considering the nature of treatment and the usual prognosis for tumors included in this study; however, the MDR bacteremia might be a significant clinical problem [10,23]. In this study, the incidence of MDR bacteria, particularly ESBL-producing *Escherichia coli*, appeared to increase over time, and four patients died from MDR bacterial infection. These findings suggest that infection from MDR bacteria might be a substantial clinical problem during HDCT/auto-SCT in the future; therefore, careful consideration in the use of antibiotics is needed to reduce the emergence of MDR bacteria.

Current guidelines for pediatric patients undergoing HDCT/auto-SCT with anticipated neutropenia for more than 7 days recommend administration of fluconazole from the start of HDCT until engraftment [24]. There were no cases of IFI in the SJCRH study using fluconazole up to 30 days post-transplant [21]. In our study, most cases received prophylactic or empirical antifungal prophylaxis and no IFI occurred. These findings suggest that the incidence of IFI in children with solid tumors might be lower than the incidence reported in adults with hematologic malignancies [25]. In this study, no antifungal agent was used in 46 (7.6%) HDCT/auto-SCTs, and there was no IFI. Collectively, these findings suggest the need to evaluate whether antifungal prophylaxis is needed in HDCT/auto-SCT for children with solid tumors.

For patients with RV infection, HDCT/auto-SCT is usually postponed until the RV infection disappears. However, in some patients, RV infection might persist for a long time without symptoms, as seen in our study. Significant delay of HDCT/auto-SCT until viral clearing might increase the risk of tumor progression, so clinicians might have to determine when to start HDCT/auto-SCT in these patients. In this study, 14 RV-positive patients without respiratory symptoms proceeded to HDCT/auto-SCT, because RV did not disappear during the waiting period. Eight of them experienced clinical respiratory infection including 2 LRTIs; however, there was no mortality. This study showed that the incidence of LRTI was relatively low (10.0%) in low-risk RV infection and there were no cases of mortality. Taken together, these findings suggest that patients with low-risk RV infection prior to HDCT/auto-SCT could proceed to HDCT/auto-SCT with relatively low-risk of severe respiratory infection, when the risk of tumor progression is high. However, for patients with high-risk RV infection, careful consideration is needed to determine when to start HDCT/auto-SCT.

VZV prophylaxis has been controversial in HDCT/auto-SCT, in contrast to allogeneic SCT [26]. Although most patients in our study did not receive VZV prophylaxis in order to avoid possible overlapping drug toxicities during HDCT, the incidence of VZV reactivation was relatively low compared to previous studies (12–26%) [27–29]. However, most patients who experienced VZV reactivation in the present study were sero-positive for VZV prior to HDCT/auto-SCT. Therefore, VZV prophylaxis might be needed for children with solid tumors who are sero-positive prior to HDCT/auto-SCT. Further study is needed.

Infectious complications other than RV infection during the late transplant period developed in 13.5% of patients. We usually removed the central venous catheter 3 or 6 months after the second HDCT/auto-SCT due to the possibility of early relapse/progression. However, most patients who experienced bacteremia during the late transplant period had indwelling central catheters at the time of infection, and most isolated organisms were Gram-positive bacteria. These findings suggest that earlier removal of central venous catheter could reduce the risk of bacteremia in the late transplant period after HDCT/auto-SCT. In addition, most infectious complications in the late transplant period occurred during the first 6 months after the second HDCT/auto-SCT; therefore, careful immune surveillance should be continued until at least 6 months post-transplant.

In summary, our study suggests that the incidence of infectious complications in our retrospective cohorts who underwent tandem HDCT/auto-SCT was lower than seen in previous reports for adults and similar to reports in other childhood cohorts, most of whom underwent single HDCT/auto-SCT. In addition, the incidence and severity of infectious complications in the second HDCT/auto-SCT was not higher than in the first HDCT/auto-SCT. However, our study is a retrospective study and diversities in disease and treatment limit our ability to draw a solid conclusion. Further study is needed to reduce the risk of infectious complications during and after tandem HDCT/auto-SCT for children with high-risk or recurrent solid tumors.
